# Prevalence and Incidence of Epilepsy Associated with Convulsive Seizures in Rural Bolivia. A Global Campaign against Epilepsy Project

**DOI:** 10.1371/journal.pone.0139108

**Published:** 2015-10-01

**Authors:** Elisa Bruno, Graziella Quattrocchi, Elizabeth Blanca Crespo Gómes, Vito Sofia, Sandra Padilla, Mario Camargo, Mario Zappia, Alessandro Bartoloni, Alessandra Nicoletti

**Affiliations:** 1 Dipartimento di Scienze Mediche, Chirurgiche e Tecnologie Avanzate “G.F. Ingrassia”, University of Catania, Via Santa Sofia 78, 95123 Catania, Italy; 2 Servicio departamental de Salud, Santa Cruz, Camiri, Bolivia; 3 President of the Bolivian League Against Epilepsy, Santa Cruz, Bolivia; 4 Department of Experimental and Clinical Medicine, Infectious and Tropical Diseases Unit, University of Florence, Largo Brambilla 3, 50134 Florence, Italy; University of Rome Tor Vergata, ITALY

## Abstract

**Objective:**

we performed a three-stages door-to-door survey to estimate incidence and prevalence of epilepsy associated with convulsive seizures (EACS) in a rural area of Bolivia.

**Methods:**

the study was carried out in the Cordillera Province, southern-eastern Bolivia. One hundred fourteen rural communities with a total population of 18,907 inhabitants were included in the survey. In order to identify subjects with EACS, trained fieldworkers administered a validated single screening question to the householders (stage I). A second face-to-face questionnaire was administered to each positive subject (stage II) that, in case of positive answer, underwent a complete neurological examination to confirm the diagnosis (stage III). We estimated age and sex specific life-time and active EACS prevalence at the prevalence day (30^th^ June 2010). Incidence risk was evaluated for the 10-year period between January 2000 and December 2010.

**Results:**

on prevalence day we identified 136 subjects with EACS, 124 of whom had active epilepsy. The life-time prevalence of EACS was 7.2/1,000 (7.6/1,000 age-adjusted to the world standard population) while the prevalence of active EACS was 6.6/1,000 (6.7/1,000 age-adjusted to the world standard population). Both life-time and active prevalence showed a peak (10.3/1,000) in the 15–24 years age group and, overall, were higher among women. During the incidence study period, 105 patients living in the study area had the onset of EACS. The crude incidence risk was 55.4/100,000 (49.5/100,000 age-adjusted to the world standard population). Incidence was slightly but not significantly higher among women (58.9/100,000 versus 51.9/100,000).

**Conclusions:**

the present study demonstrated a considerable burden of EACS in the Bolivian Chaco, showing prevalence and incidence estimates close to those reported for low and middle- income countries and underlying the need of treatment programs.

## Background

Throughout the world, epilepsy represents an important public health issue, accounting for an estimated 0.7% of the global burden of diseases [1]. Epidemiological studies are the principal instruments to identify health care priorities and to provide the background for planning primary interventions and allocating health resources. In this context, prevalence studies are largely used on a global scale. Conversely, incidence studies are more challenging, and few data are available in low-middle income countries, where the largest majority of people with epilepsy (PWE) lives [2]. At least five million PWE reside in Latin American Countries (LAC) [3], where only six incidence studies have been carried-out, resulting in an estimated median incidence of epilepsy of 138.2/100,000 persons/year (95% CI 83.6–206.4) reported in a recent meta-analysis [4]. Epilepsy is a common neurological disorder in Bolivia, where a life-time prevalence of 12.3/1,000, and an active prevalence of 11.1/1,000 were estimated in 1994, during a population-based survey performed in rural communities located in the southwestern section of Santa Cruz Department [5]. In this area, epilepsy associated with convulsive seizures (EACS) was the most frequent type (>60%) and was also associated with a high mortality and with stigma [6,7]. Moreover, a very high treatment gap (TG), near to 90%, was also reported [5]. The reduction of epilepsy TG in the area of Gran Chaco has been one of the main aims of the Global Campaign Against Epilepsy (GCAE) Project in Bolivia. This initiative, started in 2009, has the objective to set-up a valid action-plan aimed at preventing, treating and managing epilepsy at a community level in this area [7,8]. Considering that convulsive seizures are easily recognized, are associated with the highest mortality and morbidity and were identified as the most frequent seizure type in this region, the treatment program was first focused on EACS.

In order to identify PWE eligible for the treatment program, we carried-out a three-stage population-based study, estimating the prevalence and the incidence of EACS in the rural area of the Bolivian Chaco.

## Methods

### Ethics statement

The study brought together three partners: Servicio departamental de Salud, Santa Cruz, Camiri, the University of Florence and the University of Catania. The ethical committee of the University of Florence, the study coordinator, was not able to evaluate the study or to give a consent because it is not commissioned to authorize studies performed in other countries. In the absence of a local ethics committee, the study design (observational) and its ethical aspects were reviewed and approved by the National Department of Epidemiology of the Ministry of Social Welfare and Public Health and by the Guaraní political organization (Asemblea del Pueblo Guaraní, APG). All participants gave their verbal consent to take part in the study, both to the investigators and to a local language speaking anthropologist, who witnessed and documented the whole procedure. Written consent was not obtained due to illiteracy. Next of keen/guardians gave their informed verbal consent, using the same approach previously specified, in case of minors/children. Some of the interviews of participants were performed by authors of this manuscript (AN, EB, GQ, EBCG) with the help of local anthropologist (SP). The corresponding author and the first author have full access to all the information and data related to the study, including the codes used to protect confidentiality and anonymized identifying data.

### Study area

The study included all the 114 rural communities located in two municipalities (Gutierrez, and Camiri) of the Cordillera province, Department of Santa Cruz, Bolivia. The Cordillera Province covers 86,245 km^2^ and, according to the 2001 census, has a population of 101,733 inhabitants, 67,366 of which live in rural areas [9]. The two municipalities involved have a total rural population of 15,785 inhabitants [9]. During the door-to-door survey, we updated the local census at July 2010, obtaining a population of 18,907 inhabitants in the 114 rural communities. The ethnic group living in this area is mainly represented by native Guaraní people, living in poor dwellings without running water and electricity, and basing the local economy on agriculture and animal husbandry. The health-care infrastructure consists of a district hospital, 9 area hospitals, and rural health centers situated in each community, managed by nurses and local health-care personnel.

### Study design

In order to identify people with EACS we carried out a three-stages door-to-door survey from July 2010 to September 2012. Thirty-two trained local health workers performed a door-to-door screening aimed at identifying people with EACS. For the screening phase (stages I and II) we adopted a modified version of previous validated screening questionnaire [10]. The instrument was translated, adapted with the help of local anthropologists and pre-tested in the field. The questionnaire consisted of two main sections (Appendix in S1 file): the first one contained one single screening question that was administered to the householder to identify any suspected case within the family (stage I); the second section was directly administered to those people indicated as suspected cases at stage I, and included one main question (similar to the single screening question) (stage II) and five supplementary questions directed to those having answered “yes” to the first one. All people screened as positive cases (positive answer to the main question at stage II) underwent a complete neurological examination performed by specialist neurologists (stage III) to confirm the diagnosis and classify seizures type according to the clinical history.

Demographic characteristics and clinical information for all the identified people with EACS were recorded.

### Definitions of epilepsy

According to the operational definition proposed by the International League Against Epilepsy Epidemiology Commission [11,12], epilepsy was defined as a condition characterized by recurrent (at least two) seizures, unprovoked by any immediate identified cause. We defined as EACS people presenting with tonic-clonic episodes characterized by loss of consciousness lasting more than one minute, presence of tonic movements (such as generalized stiffening) and/or clonic movements (such as thrashing about) and at least one of the followings: (1) sphincter disturbance (i.e. loss of urine or stool during the fit); (2) muscle soreness after the fit; (3) fit injury (tongue biting, head cut); (4) froth coming out of the mouth; (5) falling.[13] EACS were classified as generalized convulsive or as focal onset with secondary generalization according to the simplified clinical classification of seizure type [12]. We considered as active EACS people with at least one seizure within 5 years of the prevalence day (30^th^ June 2010), regardless of AEDs treatment [11]. Furthermore, to allow comparison with other studies performed in low-income countries, we also reported the number of EACS subjects presenting at least one seizure in the 12 months preceding the prevalence day [12]. We defined as people with life-time EACS those having experienced at least two convulsive seizures in their life, before the prevalence day [11]. We considered as incident cases all the new cases of EACS occurred in the period between January 2000 and December 2010 (incidence period).

### Statistical analysis

Data were analyzed using STATA 12 software packages (version 12.0, College Station, TX). Data cleaning was performed before the data analysis considering both range and consistency checks. Quantitative variables were described using mean and standard deviation (SD). The frequency comparisons were done with the chi-square test. In case of not normal distribution, appropriate nonparametric tests were performed. Prevalence was based on the number of patients living in the study area who fulfilled the diagnostic criteria on prevalence day (30^th^ June 2010). Age and sex specific prevalence were estimated for both life-time and active EACS cases. Onset adjusted incidence risk was retrospectively evaluated for the 10-year period between January 2000 and December 2010, and separately for the two *quinquennia* (first *quinquennium* from January 2000 to December 2004; second *quinquennium* from January 2005 to December 2010). Incidence risk was based on the year of clinical onset (onset-adjusted incidence risk) and we considered as population at risk the average population, that was the mean population during the incidence study period. Confidence intervals (CIs) for estimates were calculated assuming a Poisson distribution. Crude prevalence and incidence risk were age-adjusted to the World Standard Population [14].

## Results

### Prevalence of epilepsy

**Fig 1:**
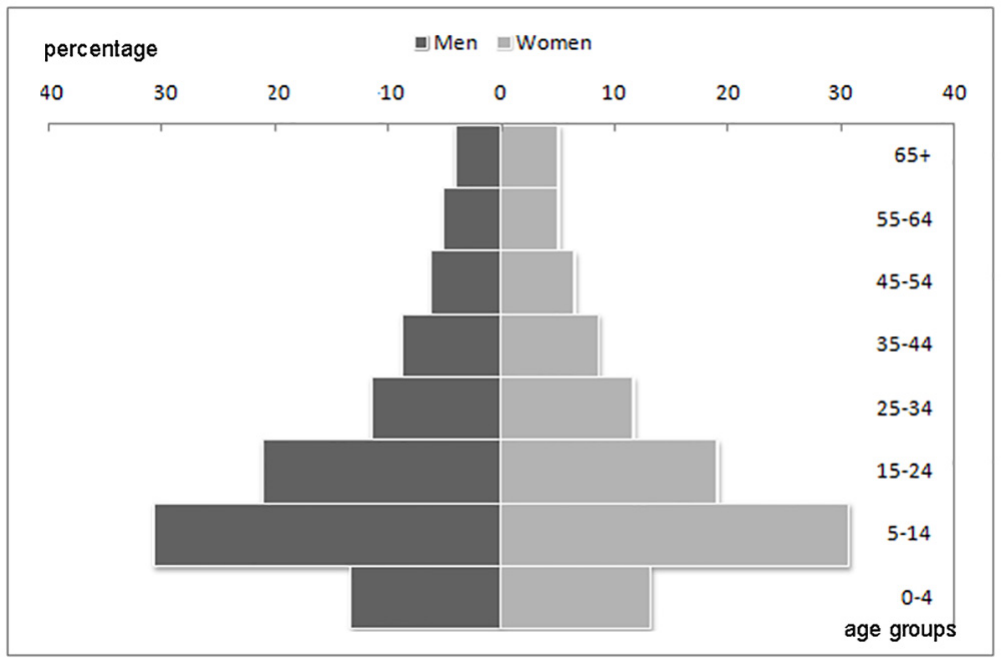
Age and sex distribution of the study population (n = 18,907).

During stage I of the screening phase we interviewed 4,155 householders in the 114 selected rural communities. The total population covered consisted of 18,907 inhabitants with a mean age of 25.9 ± 16.0 years. The sex and age distribution of the study population is shown in Fig 1.

**Fig 2:**
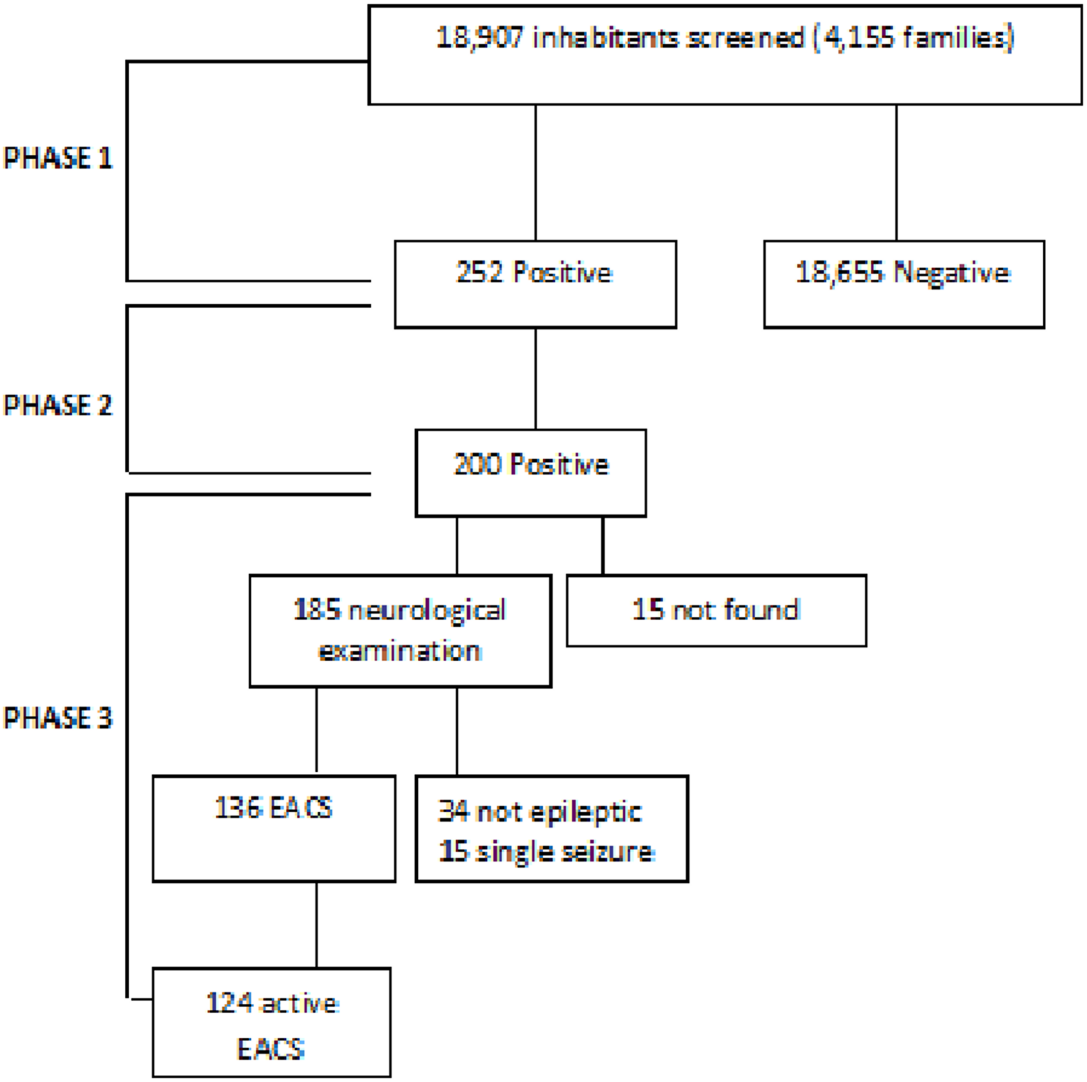
Study design and patients identification. EACS: epilepsy associated with convulsive seizures.

Two hundred fifty-two people were indicated as suspected cases at the end of stage I and underwent the supplementary questions (stage II). At the end of stage II, 200 subjects were considered positive and 185 (92.5%) were examined by neurologists at stage III (15 were not traced). Of the 185 subjects, 34 were considered not epileptic (10 syncope, 8 headache, 9 hypertension-related disturbance, 7 psychogenic non epileptic seizures), 15 had presented only a single seizure while 136 were diagnosed as EACS. We therefore detected 136 prevalent cases, 124 of whom had active epilepsy on prevalence day (Fig 2). Eighty-two people had active epilepsy considering a 12-months’ time-frame.

**Fig 3:**
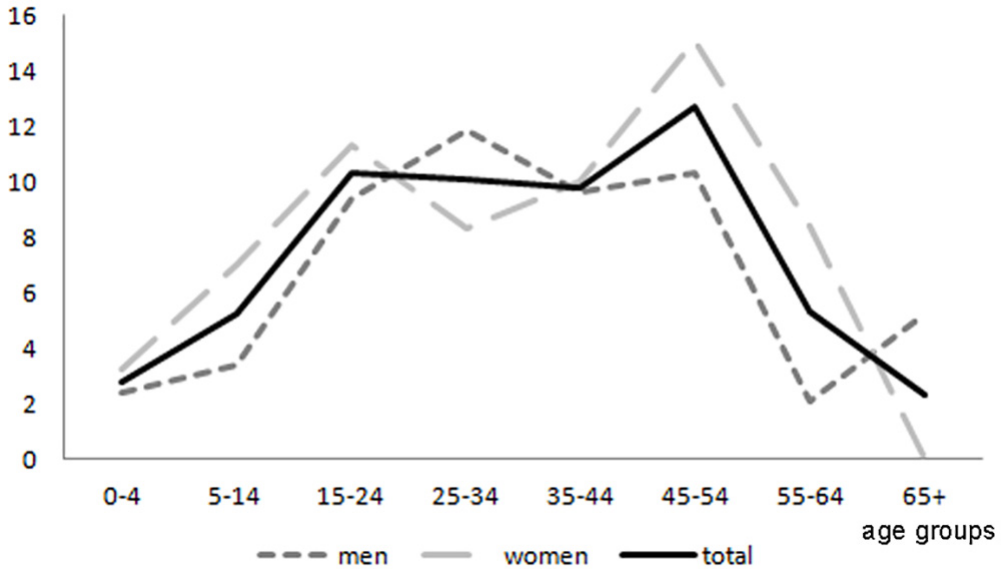
Age and sex specific life-time epilepsy associated with convulsive seizures prevalence (cases per 1,000).

**Fig 4:**
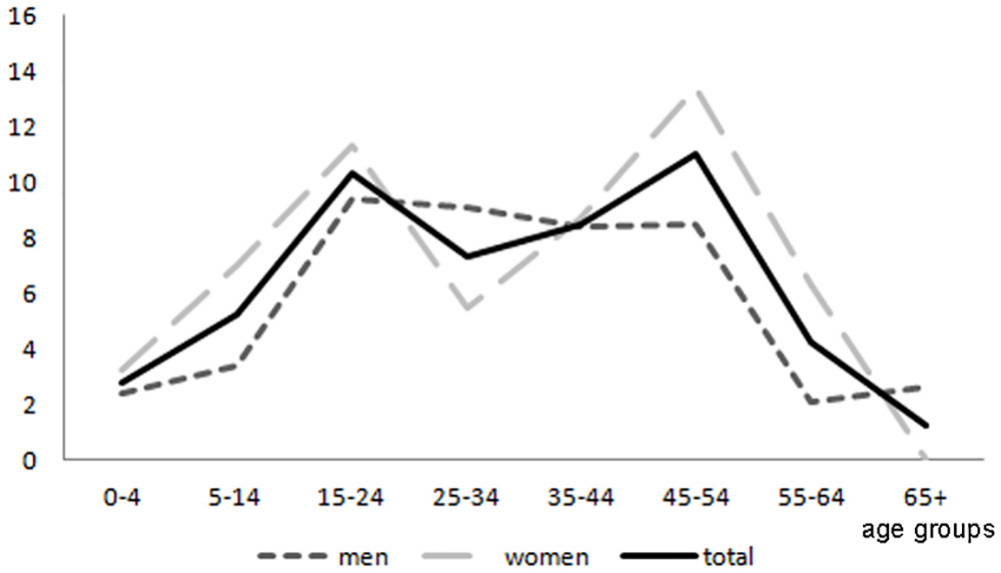
Age and sex specific active (five years definition) epilepsy associated with convulsive seizures prevalence (cases per 1,000).

The life-time EACS prevalence was 7.2/1,000 (95% CI 6.1–8.5) (7.6/1,000 age adjusted to the world standard population) and the active EACS prevalence was 6.6/1,000 (95% CI 5.5–7.8) (6.7/1,000 age adjusted to the world standard population) considering the presence of at least one seizure in the preceding 5 years, and 4.3/1,000 (95% CI 3.6–5.5) (4.2/1,000 age adjusted to the world standard population) considering the presence of at least one seizure in the preceding 12 months. Age and sex specific prevalence for life-time and active EACS are reported in Figs 3 and 4 and tables A and B in S1 file. Both life-time and active EACS prevalence showed a peak (10.3/1,000; 95% CI 7.5–14.0) in the 15 to 24-year group, declined with age, and, overall, were higher among women (life-time EACS 8.0/1,000; 95%CI 6.3–10.0; active EACS 7.3/1,000; 95%CI 5.8–9.3) than men (life-time EACS 6.5/1,000; 95% CI 5.0–8.3; active EACS 5.8/1,000; 95% CI 4.5–7.6).

According to clinical classification of seizures, of the 136 identified EACS patients, 62 (45.6%) were classified as focal onset seizures with secondary generalization and 74 (54.4%) as generalized convulsive seizures. Mean age at onset was 17.1±15.4 years for patients with focal onset with secondary generalization and 15.0±11.4 for patients with generalized convulsive seizures (p = 0.4).The mean seizures frequency was 3.0±4.8 per month for focal and 2.0±3.0 per month for generalized convulsive seizure (p = 0.1). About 87 (70.2%) identified people with active EACS was not treated with AEDs at the time of the study. Demographic and clinical characteristics are summarized in table 1.

**Table 1:** Sociodemographic and clinical characteristics of patients with epilepsy associated with convulsive seizures.

Characteristics	Focal onset seizure with secondary generalization	Generalized convulsive seizure	Total
**Number of patients** N (%)	62 (45.6)	74 (54.4)	136 (100)
**Mean age** (years±SD)	28.5±18.0	23.8±13.8	25.9±16.0
**Mean age at onset** (years±SD)	17.1±15.4	15.0±11.4	16.0±13.0
**Gender**			
Male	25 (40.3)	37 (50.0)	62 (45.6)
Female	37 (59.7)	37 (50.0)	74 (54.4)
**Marital status**			
Married	17 (27.4)	15 (20.3)	32 (23.5)
Unmarried	35 (56.5)	39 (52.7)	74 (54.4)
Not know	10 (16.1)	20 (27.0)	30 (22.1)
**Occupation**			
Farmer	8 (12.9)	13 (17.6)	21 (15.4)
Housewife	19 (30.6)	10 (13.5)	29 (21.3)
Student	16 (25.8)	19 (25.7)	35 (25.7)
Unemployed	10 (16.1)	9 (12.2)	19 (14.1)
Other	4 (6.5)	3 (4.0)	7 (5.1)
Not know	5 (8.1)	20 (27.0)	25 (18.4)
**Seizures frequency**			
Yearly	22 (35.5)	24 (32.4)	46 (33.8)
Monthly	26 (41.9)	39 (52.7)	65 (47.8)
Weekly	8 (12.9)	3 (4.1)	11 (8.1)
Daily	6 (9.7)	8 (10.8)	14 (10.3)
**Mental retardation**			
No	57 (91.9)	62 (83.8)	119 (87.5)
Yes	5 (8.1)	12 (16.2)	17 (12.5)
**Alcohol drinking**			
No	58 (93.5)	67 (90.5)	125 (91.9)
Yes	4 (6.5)	7 (9.5)	11 (8.1)
**Head trauma**			
No	52 (83.9)	62 (83.8)	114 (83.8)
Yes	10 (16.1)	12 (16.2)	22 (16.2)
**Neurological deficits**			
No	57 (91.9)	70 (94.6)	127 (93.4)
Yes	5 (8.1)	4 (5.4)	9 (6.6)
**Comorbidities**			
No	53 (85.5)	64 (86.5)	117 (14.0)
Yes	9 (14.5)	10 (13.5)	19 (86.0)
**Antiepileptic treatment**			
None	28 (45.1)	53 (71.6)	81 (59.5)
Natural remedies	13 (21.0)	3 (4.1)	16 (11.8)
Antiepileptic drugs	21 (33.9)	18 (24.3)	39 (28.7)

N: number, SD: standard deviation.

### Incidence of epilepsy

One hundred and five patients living in the study area had the onset of EACS during the incidence period. The total population was substantially stable, passing from 19,004 inhabitants in 2000 (local census) to 18,907 inhabitants in 2010. The average population in the incidence period was 18,956 inhabitants. The crude incidence risk was 55.4/100,000 (95% CI 45.1–67.4), and 49.5/100,000 when age adjusted to the world standard population. The crude incidence risk was 48.5/100,000 (95% CI 35.3–64.8) for the first *quinquennium* (2000–2004) and 62.2/100,000 (95% CI 41.8–80.1) for the second *quinquennium* (2005–2010). The risk ratio between the two periods was not significant (RR 1.3; 95% CI 0.9–1.9). Focal onset seizure with secondary generalization had an incidence of 25.0/100,000 (95% CI 18.2–33.4) and generalized convulsive seizure of 30.6/100,000 (95% CI 20.6–39.4) in the ten-year incidence period.

**Fig 5:**
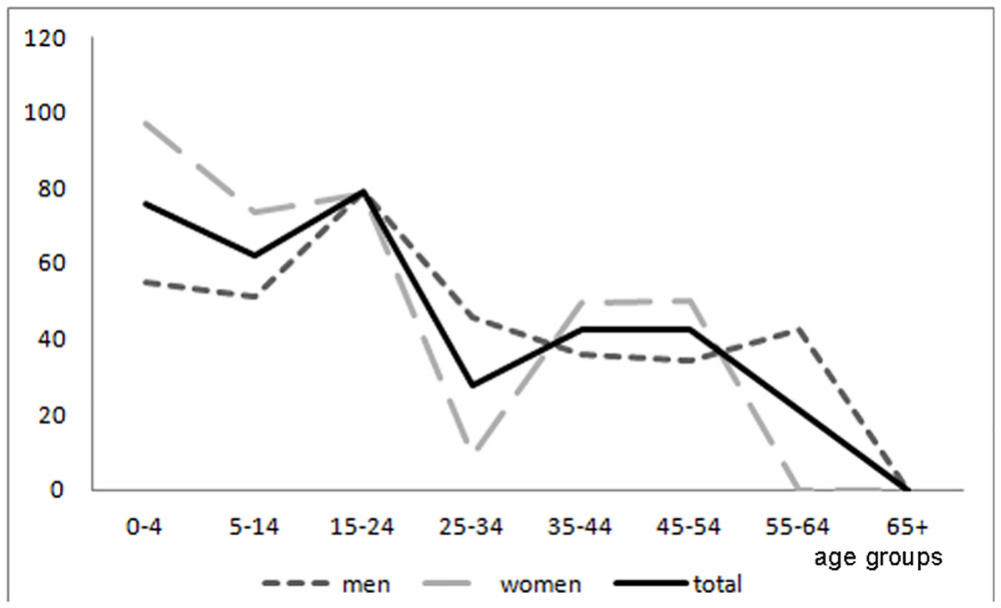
Age and sex specific incidence risk of epilepsy associated with convulsive seizures (cases per 100,000).

Age-specific incidence risk presented two peaks, the first between 0–4 years (75.6/100,000; 95% CI 45.5–118.1) and the second between 15–24 years (78.9/100,000; 95% CI 53.2–112.7) (Fig 5 and table C in S1 file). Incidence was similar in both sexes, with men presenting and incidence risk of 51.9/100,000 (95% CI 38.5–68.4) and women of 58.9/100,000 (95% CI 44.3–76.6).

## Discussion

Measures of impact and distribution of epilepsy in a specific population are an essential requirement to generate appropriate interventions and to implement disease control strategies.

This appears particularly true in low-income countries, where the scarce health resources should be appropriately and carefully allocated. Although epilepsy accounts for a large burden of the disease throughout the world [1], a wide variability of its distribution among countries has been largely demonstrated and discussed [2,15]. This suggests that epidemiological measures may be poorly generalized, as strictly associated to the demographic structure of the population and to the distribution of risk factors.

In this perspective, our study has been carried out to provide an accurate picture of the distribution of EACS in a population living in rural Bolivia to consequently develop a tailored management program.

Overall, a high burden of EACS was detected in this area. We estimated an active EACS prevalence of 6.6/1,000 (6.7/1,000 age adjusted to the world standard population) and a life-time EACS prevalence of 7.2/1,000 (7.6/1,000 age adjusted to the world standard population). This value is very close to that reported in our previous work performed in 1994 [5], where, considering both generalized tonic-clonic seizures and partial simple seizures with secondary generalization, a life-time prevalence of 9.5/1,000 was identified. Our estimates could be hardly compared to the majority of the surveys performed in Latin American Countries and, in general, worldwide, usually including both convulsive and non-convulsive seizures. A recent meta-analysis reported a median life-time epilepsy prevalence of 18.6/1,000 (95% CI 15.3–22.1) and a median active epilepsy prevalence of 13.5/1,000 (95% CI 10.2–17.2) in rural areas of Latin American Countries [4]. However, as non-convulsive epilepsy might represent up to 50% of all epilepsies in a population-based study [16,17], the whole prevalence of our population may be likely included in the above mentioned range.

Considering age-specific prevalence, we observed that both life-time EACS and active EACS showed a first peak in the 15 to 24-year group and a second peak in the 45 to 54-year group. This bimodal distribution appears narrower and slightly shifted if compared to that reported in developed countries [18,19], where the highest peaks are observed in early childhood (<15 years old) and among the elderly (>65 years old). The age-specific prevalence pattern observed in our study was akin to the inverted U-shaped distribution often seen in low and middle-income countries [16,20] and frequently related to the occurrence of belated risk factors (as infections or traumas) during life [15]. Moreover, starting from the 45 to 54-year group, prevalence estimates decreased rapidly. Tough this finding is probably influenced by the low life expectancy of this population, the occurrence of spontaneous remission of chronic epilepsy, previously demonstrated in this region [6], may have played a role.

We calculated an incidence risk of 55.4/100,000 (49.5/100,000 age adjusted to the world standard population), lower than the median incidence calculated in Latin American Countries (138.2/100,000; 95% CI 83.6–206.4), which however, is not even comparable due to the inclusion of both convulsive and non-convulsive epilepsies. The incident risk showed an apparent trend through the two *quinquennia*, tough the risk ratio between the two periods was not significant (RR 1.3; 95% CI 0.9–1.9). Considering that the occurrence of changes in risk factors in the study area is unlikely during the incidence period, the lower estimate in the first *quinquennium* may be due to a higher number of cases in remission with onset in that period, and thus, less prompt to report their condition at the time of the survey. Age-specific incidence presented a first peak among children (0–4 years) and a second among young adults (15–24 years), later declining with age and overlapping with prevalence distribution. The trend observed in early childhood should be further analyzed, also investigating the probable contribution of perinatal risk factors to this peak.

Incidence was similar in both genders (51.9/100,000 in men versus 58.9/100,000 in women), while prevalence was higher among women (life-time EACS 8.0/1,000; active EACS 7.3/1,000) than among men (life-time EACS 6.5/1,000; active EACS 5.8/1,000), as already reported in a previous survey carried-out in the same area [5] as well as in other studies performed in developing countries [21,22]. Interestingly, when the sex and age specific prevalence was considered, the life-time EACS prevalence and active EACS prevalence are largely similar among men and women till the age group 35–44. The prevalence then declines among men. Some investigations suggested that this can be an effect of higher male mortality [23]. However, we cannot exclude the effect of other socio-cultural factors and of stigma that can more likely lead men to conceal their status.

Considering seizure type, generalized convulsive seizures (54.5%) were more common than focal onset seizures with secondary generalization EACS (45.6%) in our population. The preponderance of generalized epilepsies could have been influenced by the clinical classification adopted, prone to an underestimation of those focal seizures that rapidly generalize.

A significant number of people with EACS had not received an appropriate treatment, resulting in a TG near to 70%. Even though this value still indicates a considerable number of untreated patients, it appears decreased compared to the 90% reported in our previous surveys [5,6]. This difference may be attributed to the inclusion, in the present study, of convulsive seizures only, whose severity could make people more incline to seek treatment. However, since rural health-care resources of the study area are substantially unchanged, the reduced TG could also arise from an increased awareness of the local population, stimulated by the number of activities carried-out during the past years. This enhanced treatment-seeking behavior generates good hope for the future success of the treatment program.

We are aware that one of the main limitations of the current study is its cross-sectional design, prone to recall bias (especially for seizure onset and occurrence) that might have affected both the incidence and prevalence estimates obtained. Moreover, the retrospective assessment of incident cases could have been influenced by early mortality with consequent underestimation of both age-specific and overall incidence estimates. Finally, despite the evidence of a stability of the population during the 10-year incidence period, we cannot exclude the presence of internal migration, though is often due to seasonal working displacement.

Nevertheless, the present study demonstrates a significant burden of untreated EACS in the Bolivian Chaco. It represents the background of a forthcoming program that will provide access to epilepsy care and will assure AEDs availability in a previously uncovered rural area of about 100,000 inhabitants. Applying the estimates obtained and considering that an adequate treatment could assure a normal life in 70% to 80% of patients [24], we predict that about 700 people with undiagnosed active EACS might be identified in this area and could eventually access to care, foreseeing a clinical benefit for at least 600 of them.

## Supporting Information

S1 File additional supporting information may be found in the online version of this article. **Appendix**. Screening questionnaire. **Table A**. Age and sex specific life-time epilepsy associated with convulsive seizures prevalence (cases per 1,000). **Table B**. Age and sex specific active (five years definition) epilepsy associated with convulsive seizures prevalence (cases per 1,000). **Table C**. Age and sex specific incidence risk of epilepsy associated with convulsive seizures (cases per 100,000).
(DOCX)
