# Supplementary material for: Prevalence and Incidence of Epilepsy Associated with Convulsive Seizures in Rural Bolivia. A Global Campaign against Epilepsy Project
Source: PLoS One. 2015 Oct 1;10(10):e0139108. doi: 10.1371/journal.pone.0139108 (PMC4591289; doi:10.1371/journal.pone.0139108)
Supplement: S1 File — Appendix. Screening questionnaire. Table A. Age and sex specific life-time epilepsy associated with convulsive seizures prevalence (cases per 1,000). Table B. Age and sex specific active (five years definition) epilepsy associated with convulsive seizures prevalence (cases per 1,000). Table C. Age and sex specific incidence risk of epilepsy associated with convulsive seizures (cases per 100,000). (DOCX) [file pone.0139108.s001.docx]

**S1 file**

**Appendix**

**Screening question for the householder**

Has someone of your family (included yourself) ever had a fit with loss of consciousness, without awareness of what was happening and/or uncontrolled limb movements, lasting more than one minute?

**Screening question directed to the suspected case**

Have you ever had a fit with loss of consciousness, without awareness of what was happening and/or uncontrolled limb movements, lasting more than one minute?

**Supplementary questions**

1. Did you pass urine/stool in your clothes during the episode?

2. Have you ever injured yourself or had tongue/cheek bite during the episode?

3. Was there any frothing coming from your mouth during the episode?

4. Have you ever had such an episode while asleep?

5. Do you remember what happened during the episode?

**Table A.** Age and sex specific life-time prevalence of epilepsy associated with convulsive seizures (cases per 1,000).

| **Age, years** | **Men**  **Prevalence (N); 95% CI** | **Women**  **Prevalence (N); 95% CI** | **Total**  **Prevalence (N); 95% CI** |
| --- | --- | --- | --- |
| **0-4** | 2.4 (3); 0.8-6.9 | 3.2 (4); 1.3-8.3 | 2.8 (7); 1.4-5.8 |
| **5-14** | 3.4 (10); 1.8-6.2 | 7.0 (20); 4.5-10.8 | 5.2 (30); 3.6-5.4 |
| **15-24** | 9.4 (19); 6.0-14.7 | 11.3 (20); 7.3-17.3 | 10.3 (39); 7.5-14.0 |
| **25-34** | 11.9 (13); 6.9-20.2 | 8.3 (9); 4.4-15.6 | 10.1 (22); 6.7-15.2 |
| **35-44** | 9.6 (8); 4.9-18.8 | 10.0 (8); 5.1-19.5 | 9.8 (16); 6.0-15.8 |
| **45-54** | 10.3 (6); 4.7-22.2 | 15.1 (9); 8.0-28.4 | 12.7 (15); 7.7-20.8 |
| **55-64** | 2.1 (1); 0.4-11.9 | 8.4 (4); 3.3-21.3 | 5.3 (5); 2.2-12.2 |
| **65+** | 5.2 (2); 1.4-18.7 | / | 2.3 (2); 0.6-8.5 |
| **Total** | **6.5 (62); 5.0-8.3** | **8.0 (74); 6.3-10.0** | **7.2 (136); 6.1-8.5**  **7.6*** |

*age adjusted to the world standard population

**Table B.** Age and sex specific active prevalence (five years definition) of epilepsy associated with convulsive seizures (cases per 1,000).

| **Age, years** | **Men**  **Prevalence (N); 95% CI** | **Women**  **Prevalence (N); 95% CI** | **Total**  **Prevalence (N); 95% CI** |
| --- | --- | --- | --- |
| **0-4** | 2.4 (3); 0.8-6.9 | 3.2 (4); 1.3-8.3 | 2.8 (7); 1.4-5.8 |
| **5-14** | 3.4 (10); 1.8-6.2 | 7.0 (20); 4.5-10.8 | 5.2 (30); 3.6-5.4 |
| **15-24** | 9.4 (19); 6.0-14.7 | 11.3 (20); 7.3-17.3 | 10.3 (39); 7.5-14.0 |
| **25-34** | 9.1 (10); 5.0-16.7 | 5.5 (6); 2.5-12.0 | 7.3 (16); 4.5-11.9 |
| **35-44** | 8.4 (7); 4.1-17.2 | 8.7 (7); 4.2-17.9 | 8.5 (14); 5.1-14.3 |
| **45-54** | 8.5 (5); 3.7-19.9 | 13.4 (8); 6.8-26.2 | 11.0 (13); 6.4-18.7 |
| **55-64** | 2.1 (1); 0.4-11.9 | 6.3 (3); 2.1-18.3 | 4.2 (4); 1.6-10.8 |
| **65+** | 2.6 (1); 0.5-14.6 | / | 1.2 (1); 0.2-6.6 |
| **Total** | **5.8 (56); 4.5-7.6** | **7.3 (68); 5.8-9.3** | **6.6 (124): 5.5-7.8**  **6.7*** |

*age adjusted to the world standard population

**Table C.** Age and sex specific incidence risk of epilepsy associated with convulsive seizures (cases per 100,000).

| **Age, years** | **Men**  **Incidence (N); 95% CI** | **Women,**  **Incidence (N); 95% CI** | **Total**  **Incidence(N); 95% CI** |
| --- | --- | --- | --- |
| **0-4** | 54.8 (7); 22.0-112.5 | 97.1 (12); 50.2-169.6 | 75.6 (19); 45.5-118.1 |
| **5-14** | 50.9 (15); 28.5-83.9 | 73.3 (21); 44.8-113.2 | 62.0 (36); 43.2-86.2 |
| **15-24** | 79.2 (16); 45.3-128.6 | 78.6 (14); 52.9-131.9 | 78.9 (30); 53.2-112.7 |
| **25-34** | 45.5 (5); 14.7-106.2 | 9.1 (1); 0.2-50.7 | 27.4 (6); 10.1-59.6 |
| **35-44** | 35.8 (3); 7.4-104.6 | 49.7 (4); 13.5-127.2 | 42.6 (7); 17.1-87.7 |
| **45-54** | 34.1 (2); 4.1-123.1 | 50.2 (3); 10.3-146.7 | 42.2 (5); 13.7-98.5 |
| **55-64** | 42.3 (2); 5.1-152.7 | / | 20.9 (2); 2.5-75.4 |
| **65+** | / | / | / |
| **Total** | **51.9 (50); 38.5-68.4** | **58.9 (55); 44.3-76.6** | **55.4 (105); 45.1-67.4**  **49.5*** |

*age adjusted to the world standard population
